# Outcomes after reoperated medial unicompartmental knee arthroplasties compared with primary total and primary unicompartmental knee arthroplasties: a cohort study based on local Danish databases

**DOI:** 10.2340/17453674.2025.45182

**Published:** 2026-02-03

**Authors:** Christian Bredgaard JENSEN, Claus VARNUM, Simon KORNVIG, Kristine Ifigenia BUNYOZ, Kirill GROMOV, Anders TROELSEN

**Affiliations:** 1 Clinical Orthopaedic Research Hvidovre (CORH), Department of Orthopaedic Surgery, Copenhagen University Hospital Hvidovre, Hvidovre; 2 Vejle – Centre for Orthopaedic Research (V–CORE), Deparrtment of Orthopaedic Surgery, Lillbaelt Hospital – Vejle, Vejle; 3 Department of Clinical Medicine, University of Copenhagen, Denmark

## Abstract

**Background and purpose:**

Tibial periprosthetic fractures (PPF), periprosthetic joint infections (PJI), and bearing dislocations (BD) are among the most common short-term complications in medial unicompartmental knee arthroplasty (mUKA). We aim to assess whether patients with these complications have patient-reported outcome measures (PROMs) that differ from patients with primary mUKA, primary total knee arthroplasty (TKA), or after revision TKA .

**Methods:**

This observational study included 74 mUKA patients reoperated for PPF (n = 22), PJI (n = 15), or BD (n =3 7) between January 2018 and January 2023. Comparator groups included 1,940 primary mUKA, 3,485 primary TKA, and 350 reoperated TKA patients. The primary endpoint was Oxford Knee Score (OKS) at 12 months. Missing data was imputed, and multilevel Tobit regression was used to analyze differences in PROMs.

**Results:**

At 12 months, reoperated mUKAs had lower PROM scores than primary mUKAs (OKS difference –3.3, 95% confidence interval [CI] –5.0 to –1.5) and TKAs (OKS difference –2.7, CI –4.4 to –0.9) but higher than reoperated TKAs (OKS difference: 3.0, CI 1.1 to 5.0). PPF mUKAs had 12-month scores resembling reoperated TKAs (OKS difference –0.7, CI –3.9 to 2.5). PJI mUKAs and BD mUKAs had 12-month scores resembling primary mUKAs (PJI: OKS difference –2.4, CI –6.2 to 1.5, BD: OKS difference –2.2, CI –4.7 to 0.2) and primary TKAs (PJI: OKS difference –1.7, CI –5.6 to 2.1, BD: OKS-difference –1.6, CI –4.1 to 0.8).

**Conclusion:**

Patients reoperated for PJI and BD achieved outcomes comparable to primary mUKAs and TKAs, while PPF resulted in scores lower than primary mUKAs and TKAs, comparable to reoperated TKAs.

Medial unicompartmental knee arthroplasty (mUKA) is a recommended surgical treatment for isolated anteromedial knee osteoarthritis, offering advantages over total knee arthroplasty (TKA) [1], including faster recovery, higher patient satisfaction, and better PROMs [2,3]. However, reoperations occur in 2.3% of mUKAs within 1 year, often due to tibial periprosthetic fractures (PPF), prosthetic joint infection (PJI), or bearing dislocations (BD) [4]. Understanding outcomes for these complications is essential for managing patient expectations and improving preoperative counselling.

While TKA revisions are associated with worse patient-reported outcomes compared with primary TKAs [5], these findings may not apply directly to mUKA. Complications like bearing dislocations are specific to mobile-bearing mUKA designs [6], and primary TKA components can be used in revisions from mUKA to TKA, whereas TKA revisions often require constrained implants [7]. PROMs after mUKAs revised to TKA have been shown to be either similar to or worse than primary TKAs [8,9]. In TKAs, it has been shown that PROMs after revised TKAs are impacted by the reason for revision [10], but similar knowledge in reoperated mUKAs is lacking.

The aim of our study was to investigate whether mUKA patients who experience a tibial periprosthetic fracture, PJI or bearing dislocation have PROM scores that differ from patients with a primary mUKA, primary TKA, or after reoperated TKA.

## Methods

### Study design

The study was designed as an observational study on routinely, prospectively collected data. The reporting of this study is in accordance with the Reporting of studies Conducted using Observational Routinely-Collected health Data (RECORD) Statement [11].

### Study populations

All included patients were operated or reoperated on between January 2018 and January 2023 at 2 high-volume, fast-track arthroplasty centers with a high UKA-usage (≥ 20%) in relation to TKA. Patients undergoing reoperation within this period were included, irrespective of when or where their primary procedure had been performed. The primary study group consisted of mUKA patients reoperated on for tibial periprosthetic fractures, PJIs, or bearing dislocations. The contextual comparator groups were primary mUKAs without reoperation, primary TKAs without reoperation, and reoperated TKAs.

All patients had primary knee arthroplasty for osteoarthritis, and the primary procedure comparator groups excluded those reoperated on within the study period. We defined reoperations as any surgical procedure performed due to arthroplasty complications, including soft-tissue debridement, and this definition is therefore broader than revisions. In reoperated TKA cases, all causes except secondary patellar resurfacing were included, to generate a representative TKA reoperation cohort, serving as contextual comparison. In reoperation groups only the first reoperation was included.

All included mUKAs were operated on using the mobile bearing Oxford Partial Knee (Zimmer Biomet, Warsaw, IN, USA) with a minimally invasive surgical technique with microplasty instruments. Both centers adhere to the contemporary indications for mUKA [12]: primary reduced joint space width or bone-on-bone anteromedial knee osteoarthritis, correctable deformity, functionally intact anterior cruciate ligament, and no inflammatory arthritis. The primary TKAs included in the comparator groups were operated on using either the Persona or the NexGen TKA systems (Zimmer Biomet, Warsaw, IN, USA) at 1 center and the Triathlon TKA system (Stryker, Mahwah, NJ, USA) at the other center.

### Data sources

Both centers have a local database on hip and knee arthroplasty using the Procordo software (www.procordo.com). The databases include patient characteristics, surgical data, and PROMs. Patients answered questionnaires, including PROMs preoperatively and at 3, 12, and 24 months postoperatively. Minimum questionnaire follow-up was 1 year, and maximum was 2 years. Preoperative questionnaires were filled out either at home before the preoperative physical examination or nurse-assisted at the preoperative examination. Surgeons reported surgical data postoperatively. Postoperative questionnaires were sent via email when reaching the follow-up times. If patients did not answer the questionnaire, they received a reminder via email or a phone call 2 weeks later. Patients with no reply after the reminder, or patients without an email, were sent the questionnaire in a paper version by post. We considered the data missing if no reply by email or paper version was received.

Reoperated mUKAs and TKAs were identified by reviewing the surgical schedule and the local databases on reoperated patients. We reviewed the charts of all reoperated patients to identify reasons for the reoperation, the type of reoperation, new prosthesis components, and surgical duration.

### Outcomes

The PROMs included in the local database were Oxford Knee Score (OKS) and Forgotten Joint Score (FJS). Both are developed and validated for use in knee arthroplasty [13,14]. The OKS has also been validated in revision knee arthroplasty [15]. The OKS ranges from 0 to 48 points, with 48 being the lowest pain and best function in the operated knee [14]. The OKS can be divided into poor (< 27), fair (27 to < 34), good (34 to < 42), and excellent (42–48) scores [16]. The FJS ranges from 0 to 100, with 100 being the lowest joint awareness during everyday activities following arthroplasty. The FJS is not originally meant for use preoperatively but has been used in this regard in previous arthroplasty literature to establish a baseline [17].

The primary outcome of the study was the OKS at the 12-month follow-up [18]. The secondary outcomes of the study were the OKS at 3 and 24 months’ and the FJS at 3, 12, and 24 months’ follow-up. Exploratively, we also present the proportion of OKS within the categories poor, fair, good, and excellent.

### Missing data

Complete data in PROM scores for all follow-ups were present in 50% of patients, and 81% had complete data in PROM scores at 12 months. Body mass index (BMI) was missing in 8% of patients. We imputed missing PROM scores and BMI in patients with Multivariate Imputation by Chained Equation (MICE) using Predictive Mean Matching (PMM) under the assumption of data being missing at random [19]. Missing BMI as well as, OKS, and FJS preoperatively, and at 3 months, 12 months, and 24 months, were imputed based on age, sex, BMI, type of surgery (primary mUKA/reoperated mUKA/primary TKA/reoperated TKA), and non-missing OKS and FJS. The imputation was conducted 5 times, each with 10 iterations. We present pooled mean PROM scores and BMI across all 5 imputations. PROM scores and BMI before (complete case cohort) and after imputation are presented in Table A.1 and A.2 (see Supplementary data).

### Statistics

We evaluated whether continuous variables were normally distributed using histograms and quantile–quantile plots. We presented normally distributed data using mean and SD, while non-normally distributed data was presented as median and interquartile range (IQR).

We used multilevel Tobit regression to compare PROM scores between the reoperated mUKA patients and our 3 comparator groups, taking potential ceiling effects into account [20]. A crude model with individuals as a random effect and the same model adjusted for age, sex, and BMI (see directed acyclic graph in Supplementary data) [21]) was conducted. We reported marginal means and marginal mean differences based on multilevel Tobit regression models including 95% confidence intervals (CI). Marginal means represent the model-adjusted average outcome for each group, and marginal mean differences represent the adjusted difference between groups. A significance level of 0.05 was used in all analyses.

The following contextual comparisons in primary and secondary outcomes were made using multilevel Tobit regression:

- Reoperated mUKA patients compared with the comparator groups.
- mUKA patients reoperated for tibial periprosthetic fractures compared with the comparator groups.
- mUKA patients reoperated for PJI compared with the comparator groups.
- mUKA patients reoperated for bearing dislocations compared with the comparator groups.

3 sensitivity analyses were conducted to evaluate the impact of missing data, imputation, and the use of the multilevel tobit regression model (see Table 4). The first sensitivity analysis was the multilevel Tobit regression analysis conducted on the complete case cohort (before imputation). The second and third sensitivity analyses were conventional multilevel regression models, with individuals as a random effect, conducted on the imputed cohort and the complete case cohort.

Statistical analyses were conducted using R version 4.3.0 (R Core Team, 2023; R Foundation for. Statistical Computing, Vienna, Austria) and STATA 18.0 BE (StataCorp LLP, College Station, TX, USA).

### Ethics, funding, and disclosures

Access to chart data was approved by the Regional Council of the Capital Region of Denmark (R-24011865). Data storage and retrieval of data from the local databases was approved by the Knowledge Centre on Data Protection Compliance in the Capital Region of Denmark (P-2024-17761). The local databases gathered data with consent and were approved by the Knowledge Centre on Data Protection Compliance in the Capital Region of Denmark (P-2022-290) and the Record of Data Processing Activities in the Region of Southern Denmark (17/1942). No funding was received for this study.

Complete disclosure of interest forms according to ICMJE are available on the article page, doi: 10.2340/17453674.2025.45182

## Results

**Figure 1:**
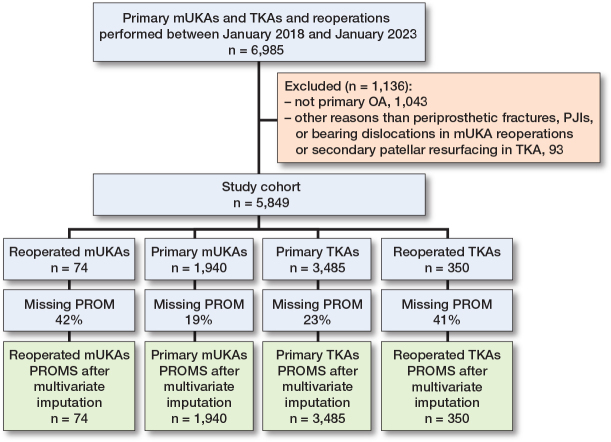
Flowchart of patients from the local databases. In the reoperated mUKA group we included only mUKA patients reoperated due to periprosthetic fracture, periprosthetic joint infection, and bearing dislocations. In the reoperated TKA group, all causes for reoperation were included, except for 13 cases of secondary patellar resurfacing. Missing PROM questionnaires is presented as the proportion (%) of missingness across all planned PROM questionnaire follow-ups within each group. OA = knee osteoarthritis, mUKA = medial unicompartmental knee arthroplasty, TKA = total knee arthroplasty, PROM = patient reported outcome measure.

We included 74 reoperated mUKAs. In the contextual comparator groups, we included 1,940 primary mUKAs, 3,485 primary TKAs, and 350 reoperated TKAs (Figure 1). Average follow-up was 2.0 years (standard deviation [SD] 0.2), with 214 patients only having 1-year follow-up. A higher proportion of women was observed in the primary TKA groups and TKA reoperations had longer surgical duration (Table 1).

**Table 1:** Patient characteristics, including preoperative patient-reported outcome measures scores for Oxford Knee Score (OKS) and Forgotten Joint Score (FJS), for primary and reoperated mUKA patients and primary and reoperated TKA patients. Values are count (%) unless otherwise specified

Factor	mUKA reoperation (n = 74)	mUKA primary (n = 1,940)	TKA primary (n = 3,485)	TKA reoperation (n = 350)
Age ^**a**^	67.0 (9.2)	66.9 (9.0)	69.4 (9.3)	68.6 (10.4)
Female sex	36 (49)	1,028 (53)	2,166 (62)	192 (55)
Body mass index ^**a**^	30 (5.3)	30 (5.5)	30 (6.1)	30 (4.8)
Surgical center				
Center 1	33 (45)	697 (36)	1,227 (35)	166 (47)
Center 2	41 (55)	1,243 (64)	2,258 (65)	184 (53)
Preoperative OKS ^**b**^	21 (17–24)	22 (17–26)	21 (16–25)	20 (16–24)
Preoperative FJS ^**b**^	14 (7–20)	13 (5–21)	12 (5–22)	14 (7–20)
Op. duration, minutes ^**a**^	63 (41)	49 (13)	67 (16)	116 (49.2)
Indication for reoperation				
Periprosthetic fracture	22 (30)	–	–	8 (2.3)
Periprosthetic joint infection	15 (20)	–	–	82 (23)
Bearing dislocation	37 (50)	–	–	0 (0)
Aseptic loosening	–	–	–	108 (31)
Pain, no loosening	–	–	–	45 (13)
Instability	–	–	–	68 (19)
Polyethylene wear	–	–	–	21 (6.0)
Other ^**c**^	–	–	–	18 (5.1)

a Values are mean (SD)

b Values are median (interquartile range)

c Other as an indication for reoperation in TKAs covered ruptured quadriceps tendons, patellar dislocation, wound dehiscence, and bone exostoses on patella.

mUKA = medial unicompartmental knee arthroplasty, TKA = total knee arthroplasty.

Median OKS and FJS for all groups and subgroups are presented in Table 2.

**Table 2:** Median Oxford Knee Scores (OKS) and Forgotten Joint Score (FJS) with interquartile ranges (IQR) for primary and reoperated mUKAs and TKAs with reoperation sorted for indication as well as treatment options in reoperated mUKAs

Factor	n	Preoperative OKS	OKS 3 months	OKS 12 months	OKS 24 months	Preoperative FJS	FJS 3 months	FJS 12 months	FJS 24 months
Primary mUKA	1,940	22 (17–26)	36 (31–41)	41 (36–45)	42 (36–45)	13 (5–21)	55 (38–73)	67 (49–83)	70 (50–84)
mUKA reoperation	74	21 (17–24)	33 (25–37)	38 (29–42)	38 (30–44)	15 (8–23)	42 (27–57)	52 (28–73)	54 (29–75)
Revised to TKA	25	20 (16–22)	29 (24–33)	33 (28–37)	32 (27–38)	9 (6–17)	35 (21–47)	39 (19–56)	38 (26–55)
Retained mUKA	49	22 (18–25)	35 (29–39)	40 (35–44)	40 (34–45)	15 (8–20)	43 (27–60)	63 (36–75)	61 (44–79)
Periprosthetic fracture	22	20 (16–23)	28 (23–34)	33 (28–37)	32 (26–38)	13 (13–22)	39 (26–48)	56 (31–75)	78 (36–81)
ORIF	6	22 (21–28)	34 (31–37)	37 (33–41)	38 (29–40)	16 (15–20)	42 (38–50)	63 (48–90)	79 (44–81)
Revision to TKA	16	19 (16–23)	28 (23–33)	32 (26–36)	32 (24–38)	17 (12–20)	40 (29–43)	52 (35–54)	72 (49–82)
Periprosthetic joint infection	15	22 (18–25)	35 (27–37)	41 (33–45)	42 (34–46)	16 (13–22)	41 (32–48)	56 (31–75)	78 (36–81)
DAIR	9	22 (19–25)	35 (24–37)	44 (35–46)	42 (35–46)	16 (15–20)	42 (38–50)	63 (48–90)	79 (44–81)
1-stage revision to TKA	3	21 (19–24)	29 (27–32)	35 (30–39)	43 (35–44)	17 (12–20)	40 (29–43)	52 (35–54)	72 (49–82)
2-stage revision to TKA	3	22 (18–23)	35 (32–39)	34 (31–38)	37 (35–41)	6 (6–25)	35 (32–56)	40 (31–59)	38 (36–58)
Bearing dislocation	37	22 (18–25)	35 (27–40)	40 (35–43)	39 (35–44)	13 (6–20)	44 (27–68)	63 (36–75)	60 (45–73)
Bearing exchange	31	22 (19–25)	36 (30–41)	40 (36–44)	41 (36–45)	13 (6–20)	48 (27–70)	65 (42–77)	60 (45–75)
Revision to TKA	6	21 (18–29)	28 (25–34)	38 (34–38)	39 (31–41)	15 (8–17)	34 (23–45)	40 (31–50)	52 (36–65)
Primary TKA	3,485	21 (16–25)	33 (27–38)	39 (33–43)	40 (35–44)	12 (5–22)	48 (29–65)	61 (41–78)	65 (45–81)
TKA reoperation	350	20 (16–24)	29 (23–33)	32 (25–37)	31 (23–38)	14 (7–20)	35 (19–50)	41 (20–58)	42 (19–61)
Periprosthetic joint infection	82	19 (17–23)	29 (25–32)	32 (26–37)	33 (27–38)	15 (9–20)	36 (25–46)	45 (31–58)	46 (34–60)
Periprosthetic fracture	8	20 (18–21)	28 (26–34)	33 (28–38)	32 (25–34)	14 (12–17)	46 (29–58)	44 (38–49)	47 (32–60)
Instability	68	20 (15–24)	29 (23–33)	30 (23–36)	28 (20–38)	15 (6–23)	28 (15–47)	35 (8–55)	40 (17–56)
Pain, no loosening	45	19 (15–22)	26 (20–31)	31 (23–36)	32 (22–38)	12 (5–18)	25 (19–40)	37 (15–58)	36 (18–59)
Aseptic loosening	108	20 (15–24)	29 (23–37)	34 (28–38)	32 (24–39)	14 (7–21)	36 (18–52)	42 (21–57)	42 (19–63)
Polyethylene wear	21	21 (17–22)	31 (28–35)	30 (25–42)	33 (20–41)	14 (4–18)	33 (21–53)	44 (15–69)	46 (14–68)
Other	18	22 (19–25)	31 (28–34)	33 (27-37)	31 (25–39)	17 (10–23)	46 (30–66)	44 (18–75)	37 (16–70)

TKA = total knee arthroplasty, mUKA = medial unicompartmental knee arthroplasty, OKS = Oxford Knee Score, DAIR = debridement, antibiotics, and implant retention (with exchange of the polyethylene bearing), ORIF = open reduction and internal fixation.

### Overall outcomes for reoperated mUKAs

**Figure 2:**
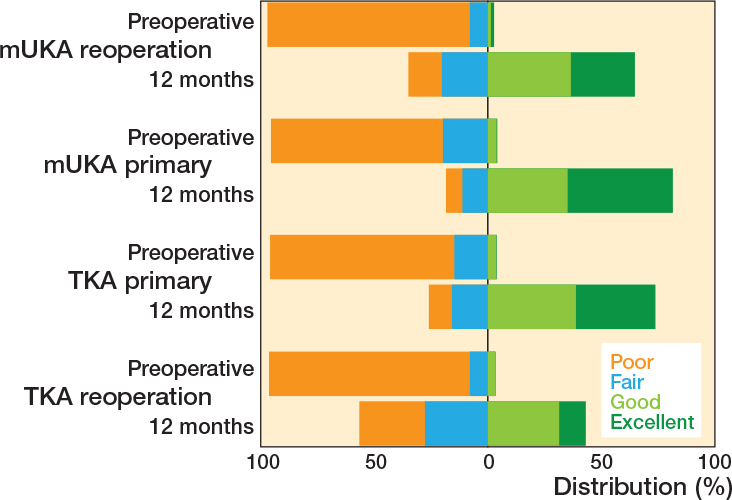
Proportion of patients achieving poor (< 27), fair (27 to < 34), good (34 to < 42), and excellent (42–48) Oxford Knee Scores within primary and reoperated mUKAs and primary and reoperated TKAs preoperatively and 12 months postoperatively. TKA = total knee arthroplasty, mUKA = medial unicompartmental knee arthroplasty.

At 12 months, reoperated mUKAs had lower PROM scores than primary mUKAs (OKS_diff_ –3.3, CI –5.0 to –1.5) and primary TKAs (OKSdiff –2.7, CI –4.4 to –0.9) but higher scores than reoperated TKAs (OKS_diff_ 3.0, CI 1.1–5.0) (Table 3). This trend persisted at 3 and 24 months. Excellent OKS at 12 months were achieved in 28% of reoperated mUKAs, compared with 47% of primary mUKAs, 35% of primary TKAs, and 12% of reoperated TKAs (Figure 2). The sensitivity analyses did not provide results leading to different conclusions; however, in the complete case cohort larger differences between groups were observed (Table 4).

**Table 3:** Oxford Knee Score and Forgotten Joint Score from Tobit regression models comparing reoperated mUKAs, periprosthetic fractures mUKAs, periprosthetic joint infection mUKAs, or bearing dislocation mUKAs with 3 comparator groups: primary mUKAs, primary TKAs, and reoperated TKAs. Estimates are reported for a crude model and a model adjusted for age, sex, body mass index, and with individuals as a random effect with 95% confidence interval

Factor	Oxford Knee Score			Forgotten Joint Score		
	mUKA primary	TKA primary	TKA reoperation	mUKA primary	TKA primary	TKA reoperation
**3 MONTHS**						
mUKA reoperation						
Crude	–2.7 (–4.5 to –1.0)	–1.2 (–2.9 to 0.6)	1.8 (0.1 to 3.7)	–14.3 (–19.8 to –8.7)	–6.1 (–11.6 to –0.7)	6.9 (0.9 to 12.8)
Adjusted	–2.7 (–4.5 to –1.0)	–1.2 (–2.9 to 0.6)	1.8 (0.1 to 3.7)	–14.3 (–19.8 to –8.8)	–6.2 (–11.7 to –0.7)	6.8 (0.9 to 12.8)
Periprosthetic fracture						
Crude	–4.7 (–7.9 to –1.5)	–3.2 (–6.3 to 0.0)	–0.2 (–3.5 to 3.1)	–20.8 (–30.8 to –10.6)	–12.6 (–22.7 to –2.6)	0.4 (–10.0 to 10.6)
Adjusted	–4.7 (–7.9 to –1.6)	–3.2 (–6.3 to 0.0)	–0.2 (–3.5 to 3.1)	–20.8 (–30.9 to –10.7)	–12.7 (–22.7 to –2.6)	0.3 (–9.9 to 10.6)
Periprosthetic joint infection						
Crude	–3.2 (–7.1 to 0.6)	–1.7 (–5.5 to 2.2)	1.3 (–2.6 to 5.2)	–18.7 (–30.7 to –6.8)	–10.6 (–22.5 to 1.3)	2.4 (–9.8 to 14.5)
Adjusted	–3.2 (–7.1 to 0.6)	–1.7 (–5.6 to 2.2)	1.3 (–2.6 to 5.2)	–18.7 (–30.7 to –6.8)	–10.6 (–22.6 to 1.3)	2.4 (–9.8 to 14.5)
Bearing dislocation						
Crude	–1.3 (–3.8 to 1.2)	0.3 (–2.2 to 2.7)	3.2 (0.7 to 5.8)	–8.6 (–16.3 to –0.8)	–0.5 (–8.2 to 7.3)	12.6 (4.5 to 20.6)
Adjusted	–1.3 (–3.8 to 1.2)	0.3 (–2.2 to 2.7)	3.2 (0.7 to 5.8)	–8.6 (–16.4 to –0.8)	–0.5 (–8.2 to 7.3)	12.5 (4.4 to 20.6)
**12 MONTHS**						
mUKA reoperation						
Crude	–3.3 (–5.0 to –1.5)	–2.7 (–4.4 to –0.9)	3.0 (1.1 to 4.9)	–16.1 (–21.6 to –10.6)	–10.4 (–15.9 to –4.9)	10.3 (4.3 to 16.3)
Adjusted	–3.3 (–5.0 to –1.5)	–2.7 (–4.4 to –0.9)	3.0 (1.1 to 5.0)	–16.1 (–21.7 to –10.6)	–10.4 (–15.9 to –4.9)	10.3 (4.3 to 16.3)
Periprosthetic fracture						
Crude	–5.6 (–8.8 to –2.4)	–5.0 (–8.1 to –1.8)	0.7 (–2.5 to 4.0)	–24.8 (–34.9 to –14.7)	–19.1 (–29.1 to –9.0)	1.6 (–8.7 to 11.9)
Adjusted	–5.6 (–8.8 to –2.4)	–5.0 (–8.1 to –1.8)	0.7 (–2.5 to 3.9)	–24.8 (–34.9 to –14.7)	–19.1 (–29.1 to –9.0)	1.6 (–8.7 to 11.9)
Periprosthetic joint infection						
Crude	–2.4 (–6.2 to 1.4)	–1.7 (–5.6 to 2.1)	3.9 (0.0 to 7.9)	–16.3 (–28.3 to –4.3)	–10.6 (–22.6 to 1.3)	10.1 (–2.1 to 22.2)
Adjusted	–2.4 (–6.2 to 1.5)	–1.7 (–5.6 to 2.1)	4.0 (0.0 to 7.9)	–16.3 (–28.3 to –4.3)	–10.6 (–22.6 to 1.4)	10.1 (–2.1 to 22.2)
Bearing dislocation						
Crude	–2.2 (–4.7 to 0.2)	–1.6 (–4.1 to 0.8)	4.1 (1.5 to 6.7)	–10.8 (–18.6 to –3.0)	–5.1 (–12.8 to 2.7)	15.6 (7.5 to 23.7)
Adjusted	–2.2 (–4.7 to 0.2)	–1.6 (–4.1 to 0.8)	4.1 (1.5 to 6.7)	–10.8 (–18.6 to –3.0)	–5.1 (–12.9 to 2.7)	15.6 (7.5 to 23.6)
**24 MONTHS**						
mUKA reoperation						
Crude	–3.8 (–5.5 to –2.0)	–3.7 (–5.5 to –2.0)	2.9 (1.0 to 4.8)	–16.9 (–22.4 to –11.4)	–12.5 (–18.0 to –7.1)	8.7 (2.7 to 14.6)
Adjusted	–3.8 (–5.5 to –2.0)	–3.7 (–5.5 to –2.0)	2.9 (1.0 to 4.8)	–16.9 (–22.5 to –11.4)	–12.5 (–18.1 to –7.1)	8.7 (2.6 to 14.6)
Periprosthetic fracture						
Crude	–7.2 (–10.3 to –4.0)	–7.1 (–10.3 to –3.9)	–0.5 (–3.7 to 2.8)	–28.6 (–38.7 to –18.5)	–24.3 (–34.3 to –14.2)	–3.1 (–13.4 to 7.2)
Adjusted	–7.2 (–10.3 to –4.0)	–7.1 (–10.3 to –4.0)	–0.5 (–3.7 to 2.8)	–28.6 (–38.7 to –18.5)	–24.3 (–34.3 to –14.3)	–3.1 (–13.4 to 7.2)
Periprosthetic joint infection						
Crude	–1.9 (–5.8 to 1.9)	–1.9 (–5.7 to 1.9)	4.7 (0.8 to 8.7)	–14.0 (–26.0 to –2.0)	–9.7 (–21.6 to 2.3)	11.5 (–0.7 to 23.7)
Adjusted	–1.9 (–5.8 to 1.9)	–1.9 (–5.7 to 1.9)	4.7 (0.8 to 8.7)	–14.0 (–26.0 to –2.0)	–9.7 (–21.6 to 2.3)	11.5 (–0.7 to 23.7)
Bearing dislocation						
Crude	–2.5 (–4.9 to 0.0)	–2.5 (–4.9 to 0.0)	4.2 (1.6 to 6.8)	–11.0 (–18.8 to –3.2)	–6.7 (–14.5 to 1.1)	14.5 (6.4 to 22.6)
Adjusted	–2.5 (–5.0 to 0.0)	–2.5 (–5.0 to 0.0)	4.2 (1.6 to 6.7)	–11.0 (–18.9 to –3.2)	–6.7 (–14.5 to 1.0)	14.5 (6.3 to 22.5)

mUKA = medial unicompartmental knee arthroplasty, TKA = total knee arthroplasty.

**Table 4:** Estimated differences in marginal mean/mean Oxford Knee Score and Forgotten Joint Score from multilevel Tobit and conventional multilevel regression models comparing reoperated mUKAs with 3 comparator groups: primary mUKAs, primary TKAs, and reoperated TKAs. The models were applied to either the full imputed cohort or the complete case cohort including only available PROM scores. Estimates are reported for crude models with individuals as a random effect and models adjusted for age, sex, and body mass index with 95% confidence interval

Factor	Oxford Knee Score			Forgotten Joint Score		
	mUKA primary	TKA primary	TKA reoperation	mUKA primary	TKA primary	TKA reoperation
**3 MONTHS**						
mUKA reoperation (imputed cohort, tobit)						
Crude	–2.7 (–4.5 to –1.0)	–1.2 (–2.9 to 0.6)	1.8 (0.1 to 3.7)	–14.3 (–19.8 to –8.7)	–6.1 (–11.6 to –0.7)	6.9 (0.9 to 12.8)
Adjusted	–2.7 (–4.5 to –1.0)	–1.2 (–2.9 to 0.6)	1.8 (0.1 to 3.7)	–14.3 (–19.8 to –8.8)	–6.2 (–11.7 to –0.7)	6.8 (0.9 to 12.8)
mUKA reoperation (complete case, tobit)						
Crude	–3.9 (–6.7 to –1.0)	–2.0 (–4.9 to 0.8)	1.4 (–1.7 to 4.5)	–13.9 (–23.4 to –4.4)	–4.5 (–14.0 to 4.9)	8.6 (–1.6 to 18.8)
Adjusted	–3.5 (–6.5 to –0.6)	–1.7 (–4.7 to 1.3)	1.8 (–1.4 to 5.1)	–13.1 (–23.0 to –3.1)	–3.8 (–13.7 to 6.1)	8.6 (–2.0 to 19.3)
mUKA reoperation (imputed cohort, conventional multilevel)						
Crude	–2.7 (–4.4 to –1.0)	–1.2 (–2.9 to 0.5)	1.8 (0.1 to 3.7)	–13.5 (–18.7 to –8.2)	–5.7 (–10.8 to –0.5)	6.4 (0.7 to 12.0)
Adjusted	–2.7 (–4.4 to –1.0)	–1.2 (–2.9 to 0.5)	1.8 (0.1 to 3.7)	–13.5 (–18.7 to –8.2)	–5.7 (–10.8 to –0.5)	6.4 (0.7 to 12.0)
mUKA reoperation (complete case, conventional multilevel)						
Crude	–3.8 (–6.6 to –1.0)	–2.0 (–4.8 to 0.8)	1.5 (–1.6 to 3.5)	–14.6 (–23.6 to –5.5)	–5.6 (–14.6 to 3.4)	6.9 (–2.8 to 16.6)
Adjusted	–3.6 (–6.5 to –0.6)	–1.8 (–4.7 to 1.2)	1.8 (–1.3 to 3.7)	–14.7 (–23.7 to –5.7)	–5.8 (–14.8 to 3.2)	6.7 (–3.0 to 16.3)
**12 MONTHS**						
mUKA reoperation (imputed cohort, tobit)						
Crude	–3.3 (–5.0 to –1.5)	–2.7 (–4.4 to –0.9)	3.0 (1.1 to 4.9)	–16.1 (–21.6 to –10.6)	–10.4 (–15.9 to –4.9)	10.3 (4.3 to 16.3)
Adjusted	–3.3 (–5.0 to –1.5)	–2.7 (–4.4 to –0.9)	3.0 (1.1 to 5.0)	–16.1 (–21.7 to –10.6)	–10.4 (–15.9 to –4.9)	10.3 (4.3 to 16.3)
mUKA reoperation (complete case, tobit)						
Crude	–3.5 (–6.3 to –0.6)	–2.7 (–4.4 to –1.0)	3.7 (0.6 to 6.7)	–13.1 (–22.5 to –3.8)	–7.2 (–16.5 to 2.2)	15.0 (4.9 to 25.1)
Adjusted	–3.5 (–6.5 to –0.6)	–2.9 (–5.9 to 0.0)	3.8 (0.6 to 7.0)	–13.1 (–22.9 to –3.3)	–7.4 (–17.2 to 2.3)	15.8 (5.2 to 26.4)
mUKA reoperation (imputed cohort, conventional multilevel)						
Crude	–3.2 (–5.0 to –1.5)	–2.9 (–4.6 to –1.2)	2.9 (1.0 to 4.7)	–13.8 (–21.1 to –6.4)	–8.4 (–15.7 to –1.1)	11.1 (2.7 to 19.5)
Adjusted	–3.2 (–5.0 to –1.5)	–2.7 (–4.4 to –1.0)	2.9 (1.0 to 4.7)	–13.8 (–21.1 to –6.4)	–8.4 (–15.7 to –1.1)	11.1 (2.7 to 19.5)
mUKA reoperation (complete case, conventional multilevel)						
Crude	–3.4 (–6.2 to –0.7)	–2.8 (–5.6 to –0.1)	3.5 (0.5 to 5.5)	–14.5 (–23.3 to –5.6)	–9.0 (–17.8 to –0.2)	13.4 (3.8 to 23.0)
Adjusted	–3.4 (–6.3 to –0.5)	–2.9 (–5.8 to 0.0)	3.7 (0.6 to 5.7)	–14.4 (–23.3 to –5.6)	–9.1 (–17.9 to –0.3)	13.3 (3.8 to 22.9)
**24 MONTHS**						
mUKA reoperation (imputed cohort, tobit)						
Crude	–3.8 (–5.5 to –2.0)	–3.7 (–5.5 to –2.0)	2.9 (1.0 to 4.8)	–16.9 (–22.4 to –11.4)	–12.5 (–18.0 to –7.1)	8.7 (2.7 to 14.6)
Adjusted	–3.8 (–5.5 to –2.0)	–3.7 (–5.5 to –2.0)	2.9 (1.0 to 4.8)	–16.9 (–22.5 to –11.4)	–12.5 (–18.1 to –7.1)	8.7 (2.6 to 14.6)
mUKA reoperation (complete case, tobit)						
Crude	–3.9 (–7.0 to –0.8)	–4.2 (–7.2 to –1.1)	3.2 (–0.1 to 6.5)	–14.1 (–24.3 to –3.8)	–10.1 (–20.3 to 0.1)	12.0 (1.1 to 23.0)
Adjusted	–3.9 (–7.1 to –0.8)	–4.4 (–7.5 to –1.2)	3.7 (–0.3 to 7.1)	–14.2 (–24.7 to –3.6)	–10.7 (–21.1 to –0.2)	13.7 (2.4 to 25.0)
mUKA reoperation (imputed cohort, conventional multilevel)						
Crude	–3.6 (–5.3 to –1.8)	–3.6 (–5.3 to –1.9)	2.9 (1.1 to 4.7)	–13.7 (–21.1 to –6.4)	–9.6 (–16.9 to –2.3)	9.9 (1.5 to 18.4)
Adjusted	–3.6 (–5.3 to –1.8)	–3.6 (–5.3 to –1.9)	2.9 (1.1 to 4.7)	–13.7 (–21.1 to –6.4)	–9.6 (–16.9 to –2.3)	9.9 (1.5 to 18.4)
mUKA reoperation (complete case, conventional multilevel)						
Crude	–3.6 (–6.6 to –0.6)	–3.9 (–6.9 to –0.9)	3.4 (–0.1 to 5.4)	–15.0 (–24.6 to –5.4)	–11.7 (–21.2 to –2.1)	11.7 (1.3 to 22.0)
Adjusted	–3.5 (–6.6 to –0.4)	–4.0 (–7.1 to –0.9)	3.9 (–0.6 to 5.9)	–14.9 (–24.5 to –5.3)	–11.6 (–21.2 to –2.0)	11.7 (1.3 to 22.0)

mUKA = medial unicompartmental knee arthroplasty, TKA = total knee arthroplasty.

### Outcomes based on mUKA reoperation indication

**Figure 3:**
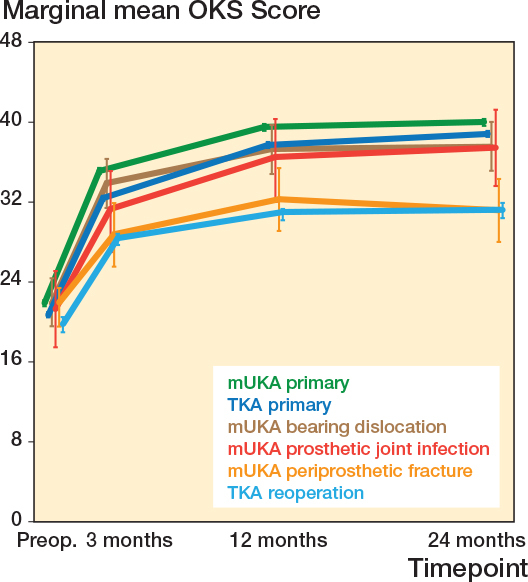
Oxford Knee Scores with 95% confidence interval (error bars) from the adjusted multilevel tobit regression models for each of the specific mUKA reoperation indications and the 3 comparator groups. TKA = total knee arthroplasty, mUKA = medial unicompartmental knee arthroplasty, OKS = Oxford Knee Score.

**Figure 4:**
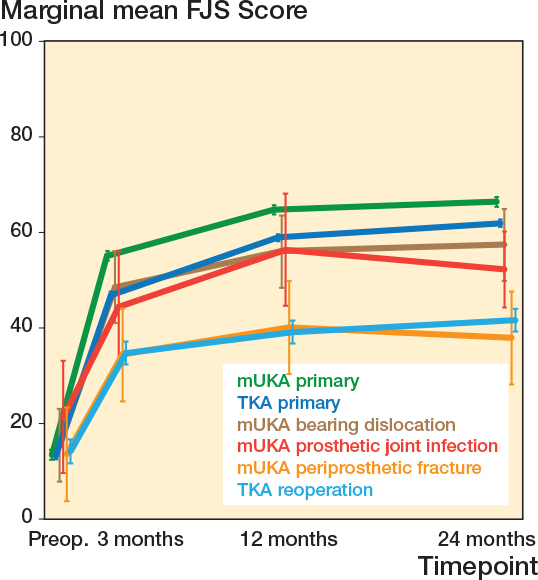
Forgotten Joint Scores with 95% confidence interval (error bars) from the adjusted multilevel tobit regression models for each of the specific mUKA reoperation indications and the 3 comparator groups. TKA = total knee arthroplasty, mUKA = medial unicompartmental knee arthroplasty, FJS = Forgotten Joint Score.

At 12 months, mUKAs reoperated on for tibial periprosthetic fractures had PROM scores comparable to reoperated TKAs (OKS_diff_ 0.7, CI –2.5 to 3.9; FJS_diff_ 1.6, CI –8.7 to 11.9). However, both primary mUKAs and primary TKAs had statistically significantly higher scores (Table 3, Figures 3 and 4).

At 12 months, mUKAs reoperated on due to PJI had PROM scores comparable to primary mUKAs (OKS_diff_ –2.4, CI –6.2 to 1.5) and primary TKAs (OKS_diff_ –1.7, CI –5.6 to 2.1), but statistically significantly higher than those of reoperated TKAs (OKS_diff_ 4.0, CI 0.0–0.9) (see Table 3, Figure 3).

At 12 months, mUKAs reoperated on for bearing dislocations had PROM scores comparable to primary mUKAs (OKS_diff_ –2.2, CI –4.7 to 0.2) and primary TKAs (OKS_diff_ –1.6, CI –4.1 to 0.8), but statistically significantly higher than those of reoperated TKAs (OKS_diff_ 4.1, CI 1.5–6.7) (see Table 3, Figure 3).

For FJS at 12 months, mUKAs reoperated on due to PJI or bearing dislocation had scores resembling primary TKAs, but statistically significantly lower than primary mUKAs (see Table 3 and Figure 4).

**Figure 5:**
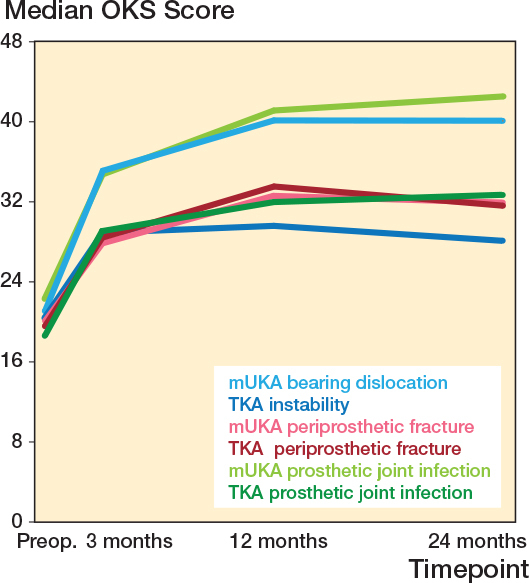
Oxford Knee Scores for reoperated mUKAs and TKAs sorted for similar indications. TKA = total knee arthroplasty, mUKA = medial unicompartmental knee arthroplasty, OKS = Oxford Knee Score.

PJI after mUKA had higher median PROM scores at 12 months compared with PJI after TKA, while mUKAs and TKAs had similar PROM scores after periprosthetic fractures (Figure 5, Table 2).

**Figure 6:**
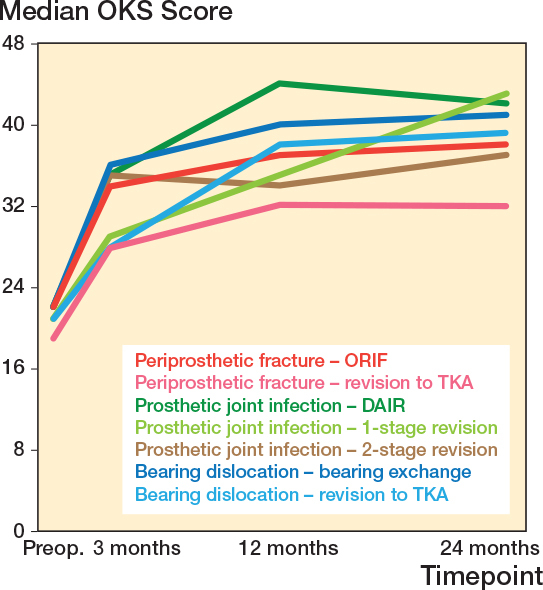
Oxford Knee Scores for reoperated mUKAs sorted for indication and treatment option. TKA = total knee arthroplasty, mUKA = medial unicompartmental knee arthroplasty, ORIF = open reduction and internal fixation, OKS = Oxford Knee Score.

Overall, reoperated mUKAs that retained their mUKA (including bearing exchange) had higher PROM scores at 12 months, compared with mUKAs revised to TKA (see Table 2 and Table 3). The least invasive treatment options for each complication resulted in the highest PROM scores (Figure 6).

## Discussion

The aim of our study was to investigate whether mUKA patients who experienced complications had PROM scores that differ from primary mUKA patients, primary TKA patients, and reoperated TKA patients. We found that mobile-bearing mUKAs reoperated on due to the most frequent short-term complications (periprosthetic fractures, PJI, and bearing dislocations) have postoperative OKS and FJS that are better than those of reoperated TKAs but worse than those of primary TKAs and primary mUKAs at 3, 12, and 24 months. Reoperation in mUKAs specifically for PJI or bearing dislocation resulted in OKS that were comparable to those of primary mUKAs and primary TKAs at 12 months and significantly higher than those of reoperated TKAs. FJS remained better in primary mUKAs compared with reoperated mUKAs for PJI or bearing dislocation.

Comparing reoperated arthroplasty patients with primary patients helps evaluate whether the outcomes can still be comparable to primary procedures despite complications. Our findings show that reoperated mUKAs achieve lower PROM scores than primary mUKAs and TKAs, but higher than reoperated TKAs. The minimal important difference (MID) for OKS is typically 5 points [22]. However, to consider smaller differences (e.g., 3 points) irrelevant is controversial. In our study, mean OKS differences for reoperated mUKAs compared with primary procedures or reoperated TKAs did not reach the MID threshold, though confidence intervals at 12 months left the possibility open. Similarly, a prior study on revised mobile-bearing mUKAs found lower PROM improvements compared with primary mUKAs and TKAs at 20 months [23]. A study on New Zealand Joint Registry data showed mUKAs revised to TKAs (mean OKS = 30.0) had similar scores to revised TKAs (mean OKS = 29.4) but lower scores than primary mUKAs (mean OKS = 39.2) and TKAs (mean OKS = 37.2) [16]. However, pain was the leading reason for revision in the mUKAs (49%), which likely contributed to poor outcomes, as unexplained pain is not a recommended revision indication [24,25].

Few studies have examined PROM outcomes after revision based on the indication [26], and to our knowledge none have reported PROMs for multiple reoperation indications in mUKA. In our study, the differences in OKS did reach the MID threshold. Similarly, a prior study on TKA revisions reported variation in OKS by revision indication, with lower scores for malalignment (24.0), stiffness (24.2), and unexplained pain (25.0), and higher scores for progressive arthritis (31.2), aseptic loosening (30.3), and fractures (29.5) [10]. While our findings for PJI in mUKAs support better outcomes after less invasive treatments, a TKA study found no significant difference in OKS between 1- and 2-stage revisions for PJI (24.9 vs 22.8) [27]. Comparing our 12-month median OKS for PJI reoperations, mUKAs outperformed TKAs (41 vs 32), emphasizing the need for dedicated studies on mUKA outcomes, as TKA data may not directly apply.

### Strengths

A strength of this study is that the mUKAs and their reoperations were performed in 2 high-volume, high-usage arthroplasty centers. Another strength is that all causes for reoperations were identified through chart review rather than diagnosis codes, ensuring greater accuracy. Common limitations of using questionnaire and PROM data include the impact of missing data and ceiling effects [28]. To address these challenges, this study utilized imputation to handle missing data and multilevel Tobit regression to account for ceiling effects in PROM scores [20]. To assess the robustness of our findings, we conducted sensitivity analyses on a non-imputed cohort, using both multilevel Tobit and a conventional multilevel regression model that did not account for ceiling effects.

### Limitations

A key limitation of this study is the small number of reoperated mUKAs for each specific complication. As a result, we did not perform statistical analyses on smaller subpopulations, such as those based on treatment options, and the data presented for these groups should be interpreted with caution. Confounding by indication is also an issue in this study when evaluating PROMs sorted for treatment options, as the complexity or severity of the complications is not accounted for but likely affects both the chosen treatment and the PROMs. The comparison of reoperated mUKAs with reoperated TKAs was purely contextual, as the primary indications, reasons for reoperation, and available treatment options differ between these groups. Providing this context helps clinicians and patients interpret the outcomes of mUKA reoperations in relation to the broader landscape of knee arthroplasty care. Additionally, we considered only the first reoperation in this study, and the potential impact of any re-reoperations was not evaluated. Missing data was particularly pronounced in the reoperation groups; however, using multiple imputation can decrease bias even in high proportions of missing data [29]. To ensure transparency, we present results based on both imputed and non-imputed data. The consistency between these analyses supports our conclusions and suggests that the observed effects are not solely an artifact of the imputation.

### Conclusion

We found that postoperative PROM scores for reoperated mUKAs vary by complication type. Reoperations for PJI and bearing dislocations achieved outcomes comparable to primary mUKAs and TKAs, while periprosthetic fractures resulted in the lowest scores, resembling those of reoperated TKAs. These findings indicate that reoperated mUKAs can achieve outcomes resembling primary knee arthroplasties, particularly with less invasive treatments.

This knowledge can be useful when discussing reoperation in mUKA patients. When comparing reoperation rates between mUKAs and TKAs, other outcomes should be considered to provide a complete understanding of their relative performance.

### Supplementary data

Tables A.1–A.2 and a DAG Figure are available as supplementary data on the article page, doi: 10.2340/17453674.2025.45182

## Supplementary Material



## Notes

CBJ and SK had full access to all data in the study and take responsibility for the integrity of the data and the accuracy of the data analysis. Study concept and design: CBJ, AT, CV, and SK. Acquisition, analysis, and interpretation of data: all authors. Drafting of the manuscript: CBJ. Critical revision of the manuscript for important intellectual content: all authors.

Handling co-editors: Li Felländer-Tsai and Philippe Wagner

*Acta* thanks Rob Nelissen and Annette W-Dahl for help with peer review of this manuscript.

## References

[1] Price A, Thienpont E, Catani F, Abram S, Troelsen A. Consensus statement on unicompartmental knee replacement: a collaboration between BASK and EKS. Knee 2023; 41 391-6. doi: 10.1016/J.KNEE.2023.03.015. 37088518

[2] Wilson H A, Middleton R, Abram S G F, Smith S, Alvand A, Jackson W F, et al. Patient relevant outcomes of unicompartmental versus total knee replacement: systematic review and meta-analysis. BMJ 2019; 364. doi: 10.1136/BMJ.L352. PMC6383371 30792179

[3] Beard D J, Davies L J, Cook J A, MacLennan G, Price A, Kent S, et al. The clinical and cost-effectiveness of total versus partial knee replacement in patients with medial compartment osteoarthritis (TOPKAT): 5-year outcomes of a randomised controlled trial. Lancet 2019; 394(10200): 746-56. doi: 10.1016/s0140-6736(19)31281-4. 31326135 PMC6727069

[4] Bredgaard Jensen C, Lindberg-Larsen M, Kappel A, Henkel C, Mark-Christensen T, Gromov K, et al. Analysis of national real-world data on reoperations after medial unicompartmental knee arthroplasty: insights from a high-usage country. Bone Joint J 2025; 107-B(3): 314-21. doi: 10.1302/0301-620X.107B3.BJJ-2024-0290.R1. 40020705

[5] Salimy M S, Paschalidis A, Dunahoe J A, Chen A F, Alpaugh K, Bedair H S, et al. Patients consistently report worse outcomes following revision total knee arthroplasty compared to primary total knee arthroplasty. J Arthroplasty 2024; 39(2): 459-65.e1. doi: 10.1016/J.ARTH.2023.08.014. 37572718

[6] Crawford D A, Berend K R, Lombardi A V. Management of the failed medial unicompartmental knee arthroplasty. J Am Acad Orthop Surg 2018; 26(20): E426-E433. doi: 10.5435/JAAOS-D-17-00107. 30113345

[7] Lunebourg A, Parratte S, Ollivier M, Abdel M P, Argenson J N A. are revisions of unicompartmental knee arthroplasties more like a primary or revision TKA? J Arthroplasty 2015; 30(11): 1985-9. doi: 10.1016/J.ARTH.2015.05.042. 26100472

[8] Lim J B T, Pang H N, Tay K J D, Chia S lu, Lo N N, Yeo S J. Clinical outcomes and patient satisfaction following revision of failed unicompartmental knee arthroplasty to total knee arthroplasty are as good as a primary total knee arthroplasty. Knee 2019; 26(4): 847-52. doi: 10.1016/J.KNEE.2019.04.016. 31113700

[9] Sun X, Su Z. A meta-analysis of unicompartmental knee arthroplasty revised to total knee arthroplasty versus primary total knee arthroplasty. Orthop Surg Res 2018; 13(1): 158. doi: 10.1186/S13018-018-0859-1. PMC6013960 29929543

[10] Sabah S A, Knight R, Alvand A, Palmer A J R, Middleton R, Abram S G F, et al. Patient-relevant outcomes following first revision total knee arthroplasty, by diagnosis: an analysis of implant survivorship, mortality, serious medical complications, and patient-reported outcome measures utilizing the National Joint Registry Data Set. J Bone Joint Surg Am 2023; 105(20): 1611-21. doi: 10.2106/JBJS.23.00251. 37607237

[11] Nicholls S G, Quach P, Von Elm E, Guttmann A, Moher D, Petersen I, et al. The REporting of Studies Conducted Using Observational Routinely-Collected Health Data (RECORD) Statement: methods for arriving at consensus and developing reporting guidelines. PLoS One 2015; 10(5): e1001885. doi: 10.1371/JOURNAL.PONE.0125620. PMC4428635 25965407

[12] Berend K R, Berend M E, Dalury D F, Argenson J-N, Dodd C A, Scott R D. Consensus Statement on Indications and Contraindications for Medial Unicompartmental Knee Arthroplasty. J Surg Orthop Adv 2015; 24(4): 252-6. 26731390

[13] Latifi R, Thomsen M G, Kallemose T, Husted H, Troelsen A. Knee awareness and functionality after simultaneous bilateral vs unilateral total knee arthroplasty. World J Orthop 2016; 7(3): 195-201. doi: 10.5312/wjo.v7.i3.195. 27004168 PMC4794539

[14] Dawson J, Fitzpatrick R, Murray D, Carr A. Questionnaire on the perceptions of patients about total knee replacement. J Bone Joint Surg Br 1998; 80(1): 63-9. doi: 10.1302/0301-620X.80B1.7859. 9460955

[15] Sabah S A, Alvand A, Beard D J, Price A J. Evidence for the validity of a patient-based instrument for assessment of outcome after revision knee arthroplasty. Bone Joint J 2021; 103-B(4): 627-34. doi: 10.1302/0301-620X.103B4.BJJ-2020-1560.R1. 33789485

[16] Pearse A J, Hooper G J, Rothwell A, Frampton C. Survival and functional outcome after revision of a unicompartmental to a total knee replacement: the New Zealand National Joint Registry. J Bone J Surg - Ser. B 2010; 92(4): 508-12. doi: 10.1302/0301-620X.92B4.22659. 20357326

[17] Clement N D, Scott C E H, Hamilton D F, MacDonald D, Howie C R. Meaningful values in the Forgotten Joint Score after total knee arthroplasty. Bone Joint J 2021; 103-B(5): 846-54. doi: 10.1302/0301-620X.103B5.BJJ-2020-0396.R1. 33934639

[18] Browne J P, Bastaki H, Dawson J. What is the optimal time point to assess patient-reported recovery after hip and knee replacement? A systematic review and analysis of routinely reported outcome data from the English patient-reported outcome measures programme. Health Qual Life Outcomes 2013; 11(1): 128. doi: 10.1186/1477-7525-11-128. 23895227 PMC3733605

[19] van Buuren S, Groothuis-Oudshoorn K. mice: Multivariate Imputation by Chained Equations in R. J. Stat. Softw 2011; 45(3): 1-67. doi: 10.18637/JSS.V045.I03.

[20] Sayers A, Whitehouse M R, Judge A, MacGregor A J, Blom A W, Ben-Shlomo Y. Analysis of change in patient-reported outcome measures with floor and ceiling effects using the multilevel Tobit model: a simulation study and an example from a National Joint Register using body mass index and the Oxford Hip Score. BMJ Open 2020; 10(8): e033646. doi: 10.1136/BMJOPEN-2019-033646. PMC7454239 32859657

[21] Hernán M A, Hernández-Diaz S, Werler M M, Mitchell A A. Causal knowledge as a prerequisite for confounding evaluation: an application to birth defects epidemiology. Am Epidemiol 2002; 155(2): 176-84. doi: 10.1093/AJE/155.2.176. 11790682

[22] Sabah S A, Alvand A, Beard D J, Price A J. Minimal important changes and differences were estimated for Oxford hip and knee scores following primary and revision arthroplasty. J Clin Epidemiol 2022; 143: 15968. doi: 10.1016/J.JCLINEPI.2021.12.016. 34920113

[23] Craik J D, El Shafie S A, Singh V K, Twyman R S. Revision of unicompartmental knee arthroplasty versus primary total knee arthroplasty. J Arthroplasty 2015; 30(4): 592-4. doi: 10.1016/j.arth.2014.10.038. 25443361

[24] Arndt K B, Schrøder H M, Troelsen A, Lindberg-Larsen M. Patient-reported outcomes and satisfaction 1 to 3 years after revisions of total knee arthroplasties for unexplained pain versus aseptic loosening. J Arthroplasty 2023; 38(3): 535-40.e3. doi: 10.1016/J.ARTH.2022.10.019. 36257505

[25] Arndt K B, Schrøder H M, Troelsen A, Lindberg-Larsen M. Patient-reported outcomes and satisfaction after revisions of medial unicompartmental knee arthroplasties for unexplained pain vs aseptic loosening. Knee Surg Sports Traumatol Arthrosc 2023; 31(11): 4766-72. doi: 10.1007/S00167-023-07483-Z. 37498328 PMC10598095

[26] Sabah S A, Alvand A, Price A J. Revision knee replacement for prosthetic joint infection: epidemiology, clinical outcomes and health-economic considerations. Knee 2021; 28 417-21. doi: 10.1016/J.KNEE.2020.12.024. 33500184

[27] Baker P, Petheram T G, Kurtz S, Konttinen Y T, Gregg P, Deehan D. Patient reported outcome measures after revision of the infected TKR: comparison of single versus two-stage revision. Knee Surg Sports Traumato Arthrosc 2013; 21(12): 2713-20. doi: 10.1007/S00167-012-2090-7. 22692517

[28] Ingelsrud L H, Wilkinson J M, Overgaard S, Rolfson O, Hallstrom B, Navarro R A, et al. How do patient-reported outcome scores in international hip and knee arthroplasty registries compare? Clin Orthop Relat Res 2022; 480(10): 1884. doi: 10.1097/CORR.0000000000002306. 35901444 PMC9473760

[29] Madley-Dowd P, Hughes R, Tilling K, Heron J. The proportion of missing data should not be used to guide decisions on multiple imputation. J Clin Epidemiol 2019; 110: 63-73. doi: 10.1016/J.JCLINEPI.2019.02.016. 30878639 PMC6547017

