# Supplementary material for: Outcomes after reoperated medial unicompartmental knee arthroplasties compared with primary total and primary unicompartmental knee arthroplasties: a cohort study based on local Danish databases
Source: Acta Orthop. 2026 Feb 3;97:67–75. doi: 10.2340/17453674.2025.45182 (PMC12869279; doi:10.2340/17453674.2025.45182)
Supplement: Supplementary file 1 [file ActaO-97-45182-s1.pdf]

SUPPLEMENTARY FIGURE AND TABLES

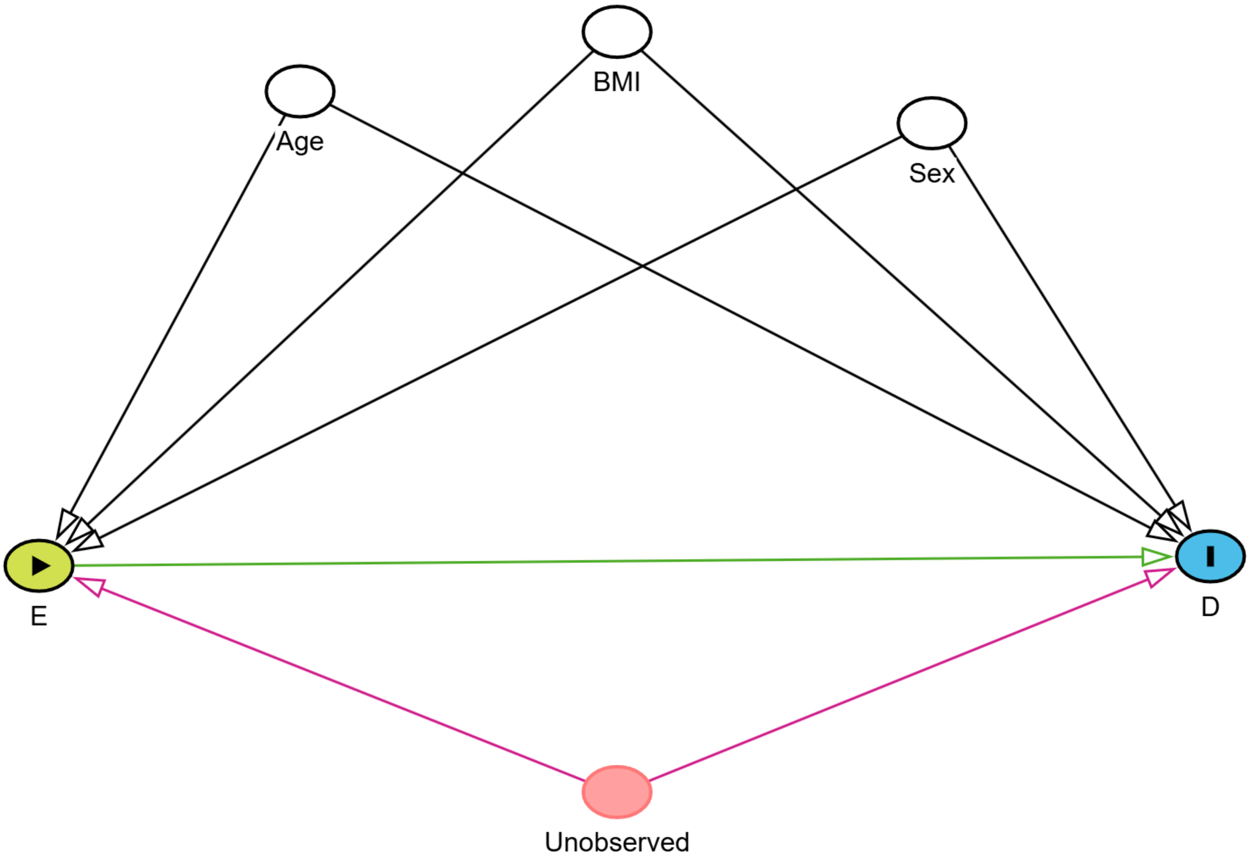

Supplementary Figure 1. Directed Acyclic Graph depicting the assumed relationship between confounding variables, age, BMI, and sex, and the Exposure (E), i.e., the type of surgery, and the Outcome (D), i.e., PROMs. Unobserved was added to openly display that we do not consider our model to account for all possible confounding factors, but only those available to us. Made using: https://www.dagitty.net/ [a].

[a] Textor J, van der Zander B, Gilthorpe M S, Liśkiewicz M, Ellison G T H. Robust causal inference using directed acyclic graphs: the R package 'dagitty'. Int Epidemiol 2016; 45(6): 1887-94, doi: 10.1093/ije/dyw341

Supplementary Table 1. Mean and standard deviation (SD) or median and interquartile range (IQR) for complete data on body mass index (BMI), Oxford Knee Score (OKS), and Forgotten Joint Score (FJS), and number of patients with missing data for each datapoint

	mUKA reoperation (n = 74)	mUKA primary (n = 1,940)	TKA primary (n = 3,485)	TKA reoperation (n = 350)
**Body mass index,** mean (SD)	30 (5.8)	30 (5.6)	30 (6.3)	30 (5.6)
Missing	14 (19)	106 (5.5)	219 (6.3)	115 (33)
**OKS preop.,** median (IQR)	20 (16–24)	22 (17–27)	20 (16–25)	20 (15–25)
Missing, n (%)	45 (61)	229 (12)	419 (12)	168 (48)
**OKS 3 months,** median (IQR)	35 (24–39)	37 (31–41)	33 (27–39)	29 (22–35)
Missing, n (%)	20 (27)	251 (13)	585 (17)	103 (29)
**OKS 12 months,** median (IQR)	40 (34–44)	42 (36–45)	40 (33–44)	32 (23–38)
Missing, n (%)	19 (26)	277 (14)	677 (19)	113 (32)
**OKS 24 months,** median (IQR)	40 (35–45)	42 (36–46)	41 (35–45)	32 (23–40)
Missing, n (%)	36 (49)	566 (29)	1,210 (35)	159 (45)
**FJS preop.,** median (IQR)	13 (0–26)	11 (4–23)	11 (4–23)	11 (4–23)
Missing, n (%)	46 (62)	325 (17)	618 (18)	185 (53)
**FJS 3 months,** median (IQR)	42 (25–67)	58 (39–75)	48 (27–67)	33 (15–52)
Missing, n (%)	21 (28)	262 (14)	623 (18)	106 (30)
**FJS 12 months,** median (IQR)	60 (29–77)	69 (48–85)	63 (40–81)	40 (15–59)
Missing, n (%)	19 (26)	282 (15)	702 (20)	121 (35)
**FJS 24 months,** median (IQR)	62 (40–79)	71 (50–88)	67 (44–83)	43 (18–70)
Missing, n (%)	38 (51)	571 (29)	1231 (35)	165 (47)

TKA = Total knee arthroplasty, UKA = medial unicompartmental knee arthroplasty, OKS = Oxford knee score, FJS = Forgotten Joint Score, preop. = preoperatively.

Supplementary Table 2. Mean and standard deviation (SD) or median and interquartile range (IQR) for body mass index (BMI), Oxford Knee Score (OKS), and Forgotten Joint Score (FJS) after imputation using Multivariate Imputation by Chained Equation (MICE) using Predictive Mean Matching (PMM)

	mUKA reoperation (n = 74)	mUKA primary (n = 1,940)	TKA primary (n = 3,485)	TKA reoperation (n = 350)
**Body mass index,** mean (SD)	30 (5.3)	30 (5.5)	30 (6.1)	30 (4.8)
**OKS preop.,** median (IQR)	21 (17–24)	22 (17–26)	21 (16–25)	20 (16–24)
**OKS 3 months,** median (IQR)	33 (25–37)	36 (31–41)	33 (27–38)	29 (23–33)
**OKS 12 months,** median (IQR)	38 (29–42)	41 (36–45)	39 (33–43)	32 (25–37)
**OKS 24 months,** median (IQR)	38 (30–44)	42 (36–45)	40 (35–44)	31 (23–38)
**FJS preop.,** median (IQR)	15 (8–23)	13 (5–21)	12 (5–22)	14 (7–20)
**FJS 3 months,** median (IQR)	42 (27–57)	55 (38–73)	48 (29–65)	35 (19–50)
**FJS 12 months,** median (IQR)	52 (28–73)	67 (49–83)	61 (41–78)	41 (20–58)
**FJS 24 months,** median (IQR)	54 (29–75)	70 (50–84)	65 (45–81)	42 (19–61)

For abbreviations, see Table.

How does patient reported outcomes after reoperated medial unicompartmental knee arthroplasties compare to primary knees?
